# Treatment Outcomes in Patients with Muscular Temporomandibular Joint Disorders: A Prospective Case-Control Study

**DOI:** 10.3390/dj12050129

**Published:** 2024-05-07

**Authors:** Rossana Izzetti, Elisabetta Carli, Stefano Gennai, Maria Rita Giuca, Filippo Graziani, Marco Nisi

**Affiliations:** 1 Department of Surgical, Medical and Molecular Pathology and Critical Care Medicine, University Hospital of Pisa, 56126 Pisa, Italy; 2 Unit of Dentistry and Oral Surgery, University Hospital of Pisa, 56126 Pisa, Italy

**Keywords:** temporomandibular joint disorders, quality of life, oral health, oral-health-related quality of life

## Abstract

Muscular temporomandibular joint disorders (M-TMDs) encompass a wide range of painful muscular conditions, which can provoke functional limitation and severely affect quality of life. The aim of the present study was to assess the treatment outcomes in patients affected by M-TMDs in terms of pain scores assessed with pressure pain threshold (PPT). The levels of depression, anxiety, and the Oral Health Impact Profile were also assessed and compared to healthy controls. Patients with a clinical diagnosis of M-TMDs and a control group of healthy subjects were enrolled. At baseline, OHIP-14, PHQ-9, and GAD-7 were administered. PPT was registered at the level of masseter and temporalis muscles. The patients affected by M-TMDs were then treated with oral splints and physio-kinesiotherapy following a standardized treatment protocol. At the 6-month follow-up of M-TMD-affected patients, PPT was registered, and the questionnaires were re-administered to evaluate treatment outcomes. In total, sixty patients and sixty controls were enrolled. The treatment of M-TMDs produced a significant improvement in PPT at the level of the masseter muscle. OHIP-14 at baseline in the M-TMD group was significantly higher compared to the control group (*p* < 0.05). At the 6-month follow-up, a significant reduction in OHIP-14 scores was registered, although with higher scores compared to the control group (*p* < 0.05). PHQ-9 was significantly higher at baseline in the M-TMD group (*p* < 0.05) and decreased to values comparable to the control group after treatment. GAD-7 presented statistically significant differences between the control group and M-TMD patients at baseline (*p* < 0.05) and between pre- and post-treatment in the M-TMD group. Following treatment, the GAD-7 scores were comparable to the control group. The symptom burden associated with M-TMDs negatively affects quality of life, with higher oral health impairment and a tendency towards depression and anxiety compared to healthy subjects. Following treatment, an improvement in both PPT and quality of life was observed.

## 1. Introduction

Temporomandibular joint disorders (TMDs) are a complex cluster of painful conditions involving both the temporomandibular joint and the masticatory muscles. The taxonomy of TMDs is defined by the Diagnostic Criteria for Temporomandibular Disorders (DC/TMD), which classify TMDs into joint disorders, masticatory muscles disorders, headache, and associated structures diseases [1].

Among the Axis I pain-related TMDs, masticatory muscles pain can be categorized as myalgia, tendonitis, myositis, and spasm [1]. Myalgia is the most encountered diagnosis, and it is reported to be among the major complaints of TMD patients [2]. Myalgia can be further divided into three subclasses, namely, local myalgia, myofascial pain, and myofascial pain with referral [1]. From a clinical point of view, it has been reported that the presentation of myalgia includes the onset of moderate pressing muscle pain, which tends to increase when provoked [3]. Masticatory function can exacerbate pain and eventually lead to difficulties in mouth opening and sharp pain [3].

In recent years, a growing interest towards patient-reported outcomes (PROs) has been developed both in research and clinical settings [4,5]. The impairment in quality of life associated with muscular TMDs (M-TMDs) in terms of pain intensity, functional limitations, and psychosocial factors has been previously reported in the literature [6,7]. Importantly, cognitive, emotional, and behavioral responses to pain have an impact on the pain dimension of quality of life in patients affected by TMDs [5].

Due to the involvement of the masticatory system, an assessment of the impact of TMDs on oral-health-related quality of life appears of utmost importance in evaluating the symptom burden directly or indirectly associated with these conditions [8,9]. Additionally, it should not be forgotten that psychological factors, including depression, anxiety, and psychological distress, can represent a risk factor for chronic pain development [10,11,12].

The aim of the present study is to evaluate the impact of M-TMDs treatment on pain, quality of life, and levels of depression and anxiety with respect to a control group of healthy subjects.

## 2. Materials and Methods

### 2.1. Study Protocol

The study was a single-center, prospective, case–control study with a follow-up after 6 months. The protocol was approved by the institutional review board of the University Hospital of Pisa (Ethics Committee North-West Tuscany area, approval no. 23815) and registered in a clinical trial database (clinicaltrials.gov, registration number NCT06339736). The study was conducted according to the principles outlined in the Declaration of Helsinki on experimentation involving human subjects. The study was reported following the STROBE statement [13].

### 2.2. Patient Enrollment

Consecutive patients referred to the Unit of Dentistry and Oral Surgery for suspected TMDs between January 2022 and December 2022 were enrolled. All of the study participants signed an informed consent form to be included in the study.

Two study groups were identified:

- M-TMD group, including patients with a diagnosis of muscular TMDs.
- Control group (CTRL) of subjects with a negative history for TMDs.

The inclusion criteria for patients in the M-TMD group were as follows: (i) males or females >18 years; (ii) systemically healthy patients; (iii) clinical examination revealing a diagnosis of M-TMD and/or positive history of M-TMD; (iv) patients willing to give informed consent; and (v) compliance to the study follow-up. Patients with (i) intraarticular joint disorders, degenerative joint disorders, arthralgia, and headache associated with TMDs, (ii) chronic systemic diseases, (iii) any psychiatric diagnosis under medication, (iv) pregnant or lactating females, and (v) not willing to comply with the study protocol were excluded.

The subjects included in the control group were enrolled among the patients referred to the Unit of Oral Surgery of the Unit of Dentistry and Oral Surgery. The patients in the control group were as follows: (i) males or females of age >18 years; (ii) systemically healthy patients; (iii) clinical examination negative for pain in the masticatory muscles and negative for history of TMDs; and (iv) patients willing to give informed consent and to be administered the study questionnaires. Patients who were (i) screened positive for TMDs, (ii) treated for systemic chronic diseases and/or psychiatric diseases, (iii) pregnant or lactating females, and (iv) not willing to comply with the study protocol were excluded.

### 2.3. Diagnosis of Muscular TMDs

Patients referred for TMDs were screened, and patients with M-TMDs (myalgia, myofascial pain, and myofascial pain with trigger point) were included. The diagnosis was performed according to the principles reported in the DC/TMD [1]. Briefly, clinical examination involved the palpation of the temporalis and masseter muscles bilaterally, with an initial pressure of 1 Kg for 2 s to rule out the presence of M-TMD. Subsequently, the confirmation of pain and the spreading of pain or referral were evaluated by increasing pressure time to 5 s. The clinical assessment of all the patients included in the study was performed by a single examiner expert in TMDs and orofacial pain (intraclass correlation coefficient >0.9 for the assessment of intra-examiner reliability).

### 2.4. Pressure Pain Threshold Evaluation

The pain evoked by muscular palpation was assessed through pressure pain threshold (PPT) with the support of a dial algometer (Wagner Pain Test™ FPX; Wagner Instruments, Greenwich, CT, USA). A 1 cm^2^ pressure was applied on the sites examined through palpation (masseter superior, masseter middle, masseter inferior, temporalis anterior, temporalis middle, and temporalis posterior) [14]. All the measurements were performed bilaterally. Assessment was performed at baseline in M-TMD-affected patients and in controls. PPT registration was repeated in M-TMD patients at six months follow-up.

### 2.5. Treatment of Patients with Muscular TMDs

After diagnosis confirmation of M-TMDs according to the DC/TMD criteria, the patients were treated with the application of an oral splint on the upper dental arch. Specifically, a stabilization appliance was employed, as it is the most recommended for the treatment of muscular disorders [15,16]. This type of splint is applied to the maxillary dental arch and is characterized by a balanced occlusion in the musculo-skeletally stable position, canine guidance, heavier contact on the posterior teeth compared to anterior teeth during closure, and a flat occlusal surface. The splint was prescribed for nighttime use in all patients and additional daytime use initially in patients needing physical self-regulation.

The treatment with the oral appliance was associated with physio-kinesiotherapy, which involves muscular stretching and relaxation exercises and then strengthening and endurance exercises to achieve stabilization. An initial assessment was performed by a physiotherapist expert in M-TMDs management (intraclass correlation coefficient >0.9 for the assessment of intra-examiner reliability), and 10 treatment sessions were scheduled. The exercises were prescribed following the 6x6 scheme by Rocabado, involving 6 exercises to be performed 6 times a day, to be repeated 6 times each [17]. Briefly, the exercises were as follows:

1. Tongue repositioning on the palate behind the incisors.
2. Correction of shoulder posture through shoulder girdle retraction.
3. Head stabilization through distraction of the upper cervical spine.
4. Axial extension of the neck through distraction of the cervical vertebrae.
5. Reduction in initiating jaw movements with translatory component to improve the control of TMJ rotation.
6. Induction of muscle relaxation through the principle of reciprocal inhibition with rhythmic stabilization technique.

The patients were then re-evaluated by both the dentist and the therapist six months after the end of the physio-kinesiotherapy and checked for complete remission of symptoms.

### 2.6. Assessment of Patient-Reported Outcomes

At the time of the enrollment, both M-TMD patients and controls were administered three questionnaires assessing oral health, depression, and anxiety.

The Oral Health Impact Profile (OHIP) Questionnaire was designed by Slade and Spencer [18] to evaluate the social impact of oral disorders through self-reporting. The original version encompassed 49 questions, although a shortened 14-item OHIP questionnaire was also devised [19]. The OHIP-14 questionnaire investigates various aspects of oral health assessed through seven domains, specifically, functional limitations, physical pain, psychological discomfort, physical disability, social disability, and handicap, endorsed on a 5-point Likert scale ranging from never (score 0) to very often/every day (score 4) [19]. The questionnaire scores thus range between 0 and 56, as the sum of the ordinal values attributed to each item. A higher OHIP-14 score corresponds to worse oral health.

The Patent Health Questionnaire-9 (PHQ-9) was developed by Spitzer et al. [20] as a tool to simplify the assessment of depression. It comprises 9 questions investigating the occurrence of depression symptoms in the previous two weeks on a scale ranging from “not at all” (score 0) to “nearly every day” (score 3), which allows for the assessment of depression severity [20]. The final score is calculated as the sum of the values attributed to each item, with a score ranging between 0 and 27. The following diagnosis is performed according to the score obtained: 0–4, none–minimal depression; 5–9, mild depression; 10–14, moderate depression; 15–19, moderately severe depression; 20–27, severe depression.

The Generalized Anxiety Disorder-7 (GAD-7) Questionnaire was developed to assess anxiety symptoms severity and to monitor changes over time [21]. The questionnaire is composed of 7 items, which can be scored from 0 to 3 (0: not at all, 1: several days, 2: more than half the days, and 3: nearly every day), for a total scale ranging between 0 and 21. Cut points of 5, 10, and 15 are set to discriminate between different levels of anxiety; specifically, 0–5 refer to mild anxiety, 6–10 to moderate, 11–15 to moderately severe anxiety and 16–21 to severe anxiety. Both the PHQ-9 and GAD-7 have been previously employed for the assessment of depression and anxiety levels in TMD-affected patients [22].

### 2.7. Sample Size Estimation

The aim was to evaluate the differences in PPT, OHIP-14, PHQ-9, and GAD-7 in M-TMD patients compared to control subjects. Since the change in the scores was the primary outcome measure, an estimate of the sample size was made using the following assumptions: significance level a = 0.05, power = 0.9, and difference in proportion = 0. These hypotheses required a sample size of at least 57 subjects (per group) to obtain valid and reliable results, capable of detecting a significant difference.

### 2.8. Statistical Analysis

All values are presented as mean (SD). PPT values pre- and post-treatment were compared with Student’s *t*-test. A comparison between OHIP-14, PHQ-9, and GAD-7 values at baseline and at follow-up in M-TMD patients was performed using the Mann–Whitney test. The baseline and follow-up values of the questionnaires were further compared with respect to the control group. The *p*-value was set for *p* < 0.05.

## 3. Results

### 3.1. Population Characterisitcs

In total, 120 patients (60 affected by M-TMDs and 60 controls) were enrolled. The M-TMD group was composed of 34 females and 26 males (mean age 40.71, SD 15.85). The control group was composed of 32 females and 28 males (mean age 42.76, SD 16.94). No statistical differences were present among groups in terms of age and gender distribution. All the subjects completed the study.

### 3.2. PPT Assessment

**Figure 1:**
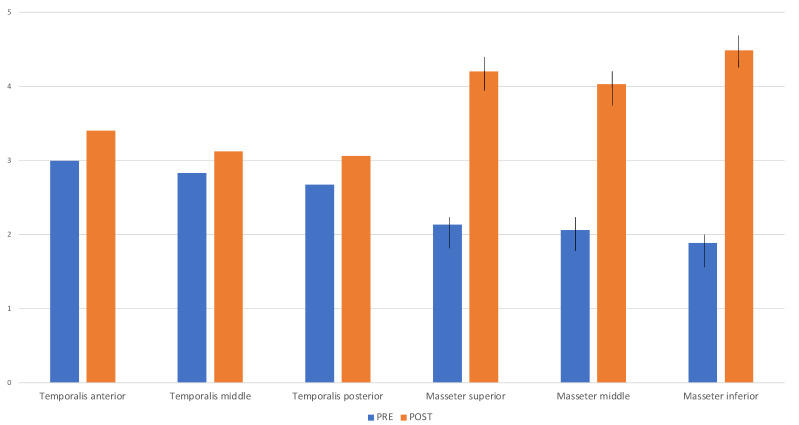
Pressure pain threshold variation between pre- and post-treatment in patients affected by M-TMDs.

At baseline, the M-TMD group showed lower PPT values for the masseter muscle compared to the temporalis muscle. Masseter inferior showed the lowest PPT value (1.88, SD 1.20). At the 6-month follow-up, significant increases in PPT values were registered for the masseter muscle in all its portions (*p* < 0.05). For the temporalis muscle, no statistically significant differences were detected in the M-TMD group between baseline and 6-months follow-up. Comparison of M-TMD pre-treatment with control group highlighted a statistically significant difference in PPT scores for the masseter muscle (*p* < 0.05) (Table 1, Figure 1).

### 3.3. Questionnaire Scores

**Figure 2:**
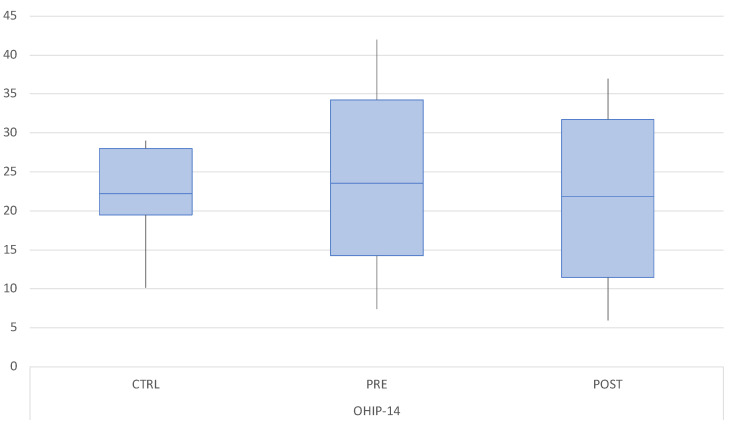
Boxplot of the mean OHIP-14 scores in the control group (CTRL) at baseline and in the M-TMD group before and after treatment.

The mean OHIP-14 score in the M-TMD group at baseline was 20.44 (SD 12.06). This value was significantly higher compared to the control group (6.77, SD 5.85, *p* < 0.05). Following treatment, OHIP-14 significantly decreased to 15.18 (SD 11.49) (*p* < 0.05). OHIP-14 in the TMD group remained significantly higher than the score registered in the control group (*p* < 0.05) (Figure 2).

**Figure 3:**
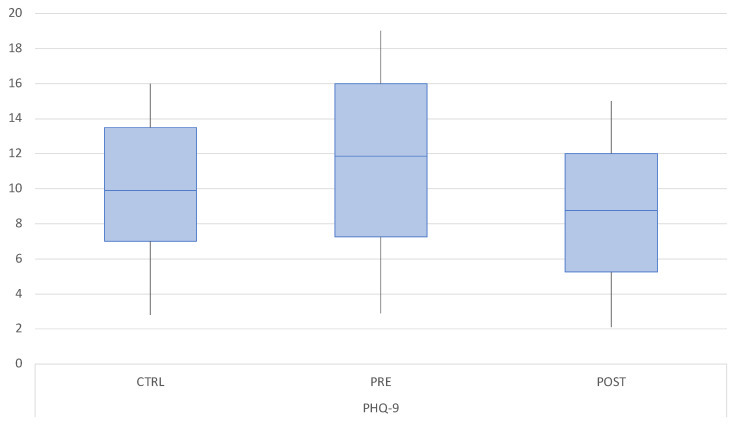
Boxplot of the mean PHQ-9 scores in the control group (CTRL) at baseline and in the M-TMD group before and after treatment.

The mean PHQ-9 score was 6.10 (SD 4.54) in the control group, 7.14 (SD 5.44) in the M-TMD group pre-treatment, and 6.24 (SD 4.56) at follow-up. PHQ-9 was significantly higher in M-TMD patients before treatment compared to the control group and to post-treatment. No differences between the control group and M-TMD group post-treatment were registered (Figure 3).

**Figure 4:**
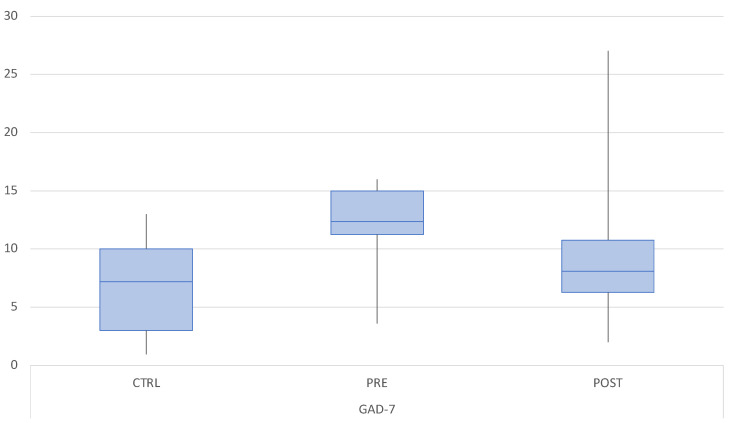
Boxplot of the mean GAD-7 scores in the control group (CTRL) at baseline and in the M-TMD group before and after treatment.

The mean GAD-7 score was 4.65 (SD 4.06) in the control group, 13.79 (SD 5.39) in the M-TMD group pre-treatment, and 6.91 (SD 3.42) at follow-up. GAD-7 was significantly lower in the control group compared to M-TMD patients at baseline (*p* < 0.05). No differences were registered between the control group and M-TMD group post-treatment (Figure 4).

The mean scores obtained for the administered questionnaires in the control group and in the M-TMD group pre- and post-treatment are reported in Table 2.

## 4. Discussion

The present results corroborate the hypothesis that patients affected by M-TMDs present an overall impairment in quality of life, with higher levels of oral discomfort and a tendency to anxiety and depression. It appears worth noting that M-TMD patients consistently presented higher OHIP-14, PHQ-9, and GAD-7 scores at baseline compared to the control group. Although the treatment protocol proved effective in reducing the pressure pain threshold in M-TMD-affected patients compared to baseline, OHIP-14, PHQ-9, and GAD-7 scores still maintained higher values compared to non-affected patients, suggesting that the symptom burden related to M-TMDs may affect the patient at a deeper level that is only partially solved by the treatment.

A recent network meta-analysis by Al-Moraissi et al. [3] highlighted the lack of consensus regarding the most effective treatment for M-TMDs. The authors found that manual therapy was the most effective treatment for M-TMDs, followed by counseling, local anesthesia, and occlusal appliances, although with a low quality of evidence. Occlusal appliances and manual therapy are the therapeutic approaches with a superior treatment effect in the short and intermediate term [3]. Previous evidence from the literature highlighted that oral splints provide a stabilizing effect through mandibular repositioning [23]. However, the actual mechanism behind this positive therapeutic effect appears to still be debated. It has been hypothesized that various factors, including the achievement of an orthopedically stable position, the reduction in masticatory muscle activity and joint loading, and the change in the temporomandibular joint functional relationship, may all concur to an overall improvement in M-TMD symptoms, although highlighting the need for close monitoring to prevent the onset of adverse effects [23].

In our sample, a combined approach involving the application of oral splints with physio-kinesiotherapy sessions appeared effective in reducing muscular pain. This beneficial effect of physiotherapy entails the recovery of normal joint movements and a reduction in pain through an increase in muscle coordination, the normalization of the range of motion, muscular relaxation, and the increase in muscle strength [24,25]. The physiotherapeutic approach involved the prescription of both stretching and strengthening exercises, to improve muscular coordination and decrease tension of the muscular fibres. This approach has been reported to provide an improvement of M-TMDs symptoms, despite the presence of variable protocols in the literature and the need for a comprehensive knowledge of joint biomechanics and muscular anatomy [24]. Our results thus appear consistent with current evidence, although the combined approach involving oral splints and physiotherapy hinders to evaluate the actual benefit deriving from each treatment.

The increase in PPT following treatment represents a relevant treatment outcome due to the relationship between this parameter and the severity of signs and symptoms of M-TMDs [26]. It has been previously reported that patients with M-TMDs experience lower PPT values at the level of the temporalis and masseter muscles compared to healthy patients, presumably in light of the central sensitization caused by chronic pain in M-TMD patients accounting for a reduction in muscle pain threshold [4]. Moreover, healthy patients experience higher PPT at the level of the masseter muscles compared to patients with M-TMDs, even when the assessment is carried out during forced muscular contraction with maximum occlusal intercuspation [27]. It should be mentioned that other factors, including somatosensory amplification, may act as confounders in the assessment of M-TMD patients while evaluating the response to masticatory muscle palpation [28]. Due to the presence of such variability, the evidence available in the literature regarding the effects of different treatment approaches on PPT is still controversial. Stabilization splints have been reported to promote symptom remission and functional reestablishment as confirmed by means of diagnostic imaging [29]. However, evidence also suggests an improvement in PPT following treatment with stabilization splints and manual therapy when compared to oral splints alone, with stable results at 1-month follow-up [30]. However, a recent systematic review highlighted that positive effects on the signs and symptoms of M-TMDs can be observed following different treatment approaches, despite insufficient evidence allowing for the determination of which treatment is more beneficial [31]. Overall, it seems that occlusal splint therapy and exercise therapy both contribute to pain relief and improvement in mandibular movement for painful TMD patients, with the lack of a superiority of exercises over occlusal splints [32]. The meta-analysis by Kuzmanovic Pficer et al. [33] isolated the effect of stabilization splints from other treatment approaches and highlighted an improvement in pain symptoms in the short term, while the effects tended to decrease on the long term. Overall, the preferred treatment approach still seems to be open for debate in light of the present literature, highlighting the unmet need for further assessment through randomized clinical trials with long term follow-up.

Notably, in our sample, an improvement in PPT was found only in the masseter muscle. This fact may be ascribed to an overall decrease in parafunctional activity following the application of the stabilization appliance along with muscular relaxation exercises. Moreover, during closure on the stabilization appliance, the functional pull of the masseter helps position the condyles in their most supero-anterior position at the base of the posterior slopes of the articular eminences [34]. Therefore, all these factors may be recognized to concur to an overall and more evident increase in PPT of the masseter muscle. Indeed, it should be borne in mind that in patients affected by M-TMDs, pain intensity may not be sufficiently reliable in the assessment of treatment efficacy due to the interference of other physical, psychosocial, and behavioral factors [35]. Treatment responsiveness thus appears to be more complex than just pain intensity, as it also reflects patient satisfaction, physical functioning, and psychosocial factors. The connection between orofacial painful conditions and an impairment in quality of life has been extensively reported in the literature. TMDs are defined as chronic painful conditions, which present as dominant characteristic the persistence of pain past normal healing time [36]. Chronic pain of moderate to severe intensity occurs in almost 20% of adults and seriously affects the quality of social and working life [37]. Patients affected by conditions related to orofacial pain report a moderate impact for the pain dimension of oral-health related quality of life, although data on pain should always be integrated with oral function, orofacial appearance, and psychosocial impact [3].

Psychosocial aspects play a relevant role in patients affected by TMDs and are defined within Axis II of DC/TMD, which establishes the screening methodology for pain intensity, psychosocial distress, and pain-related disability assessment [1]. The introduction of the biobehavioral domain by the RDC/TMD [7] finds its rationale in the fact that the observed levels of pain and disability cannot always be related to the actual clinical diagnosis, with symptoms of depression and/or anxiety negatively affecting the clinical course of TMDs [38]. However, it is also recognized that depression and anxiety are among the risk factors for chronic pain onset in musculoskeletal disorders [39]. Previous evidence supports the development of higher levels of psychological and affective distress, greater perceived stress and catastrophizing, and increased somatic awareness in patients affected by TMDs versus controls, further supporting a predisposing role of biobehavioral factors in the development of chronic pain [40].

In our cohort, the psychological profile assessed through PHQ-9 and GAD-7 highlighted higher levels of depression and anxiety in M-TMD patients, which remained higher compared to control group even after treatment. These findings are consistent with the previous literature highlighting the presence of higher levels of Axis II scores irrespective of pain duration and pain intensity [41]. Importantly, the psycho-affective profile may contribute to the persistence of the symptoms in M-TMDs and should be further assessed to better understand treatment implications and outcomes in these patients. Additionally, M-TMDs can worsen the quality of life, especially in patients with high self-reported pain or pain catastrophizing [4]. This latter aspect represents an extremely complex phenomenon, which can be characterized as an exaggerated perceived threat of pain sensation while enclosing a multidimensional construct [42]. Pain catastrophizing encompasses phenomena of rumination, magnification, and helplessness, which can eventually lead to kinesiophobia as a consequence of the fear of pain [4]. It appears extremely relevant that patients affected by M-TMDs have an 8.673 times higher chance of having a poor quality of life, as a result of the association between TMD, pain, and pain catastrophizing [43]. Similarly, patients with myofascial pain experience higher levels of anxiety and depression compared to patients with other subtypes of TMDs [44,45]. Our results confirm the presence of a reduced quality of life as assessed through OHIP-14 in patients affected by M-TMDs. Following treatment, OHIP-14 scores improved to an extent that is considered highly relevant for the patient [46].

This study has some limitations. The M-TMD group was treated with a combined approach, which hindered the assessment of the actual impact of the oral splint versus physio-kinesiotherapy in M-TMDs. Although the aim of the study was to assess how treatment impacted pain, quality of life, and levels of depression and anxiety in M-TMD-affected patients compared to healthy controls, it was not possible to assess the superiority of the treatment approach involving the use of stabilization appliances compared to physio-kinesiotherapy. While we recognized that combined treatment was overall effective, separate assessment of oral splints versus physio-kinesiotherapy is advised to evaluate the weight of the two techniques in managing M-TMDs. Moreover, counseling was not performed and the role of stressful life events experience as a causative factor was not assessed in the present cohort. Finally, the assessment of patient-reported outcomes still represents a potential source of bias due to the subjectivity of questionnaires interpretation.

Nevertheless, the present study highlights how a combined treatment approach can prove beneficial in both reducing pain symptoms associated with M-TMDs and improving overall quality of life. It appears that the symptom burden of M-TMDs may also be affected by a strong emotional component, thus contributing to a psychological impact on quality of life. Further studies on larger samples are needed to more deeply investigate the characteristics of pain provoked by M-TMDs.

## 5. Conclusions

The combined treatment with oral splints and physio-kinesiotherapy appears beneficial in reducing pain symptoms associated with M-TMDs. An overall improvement in OHIP-14 scores and in the levels of depression and anxiety can be observed following treatment, highlighting the need to better assess the psychological aspects involved in the course of M-TMDs. The present study lays a foundation for further work that could explore different comparisons, which may shed more light on this topic.

## Figures and Tables

**Table 1:** Mean PPT scores in the control group (CTRL) at baseline and in the M-TMD group before and after treatment. All the values are expressed as mean (standard deviation).

Muscle	CTRL	M-TMD PRE	M-TMD POST	CTRL vs. PRE	CTRL vs. POST	PRE vs. POST
Masseter inferior	4.12 (0.98)	1.88 (1.20)	4.48 (1.18)	*p* < 0.05	NS	*p* < 0.05
Masseter middle	4.52 (0.88)	2.06 (1.24)	4.03 (1.26)	*p* < 0.05	NS	*p* < 0.05
Masseter superior	4.67 (0.93)	2.13 (1.15)	4.20 (1.24)	*p* < 0.05	NS	*p* < 0.05
Temporalis anterior	3.02 (0.75)	2.99 (0.97)	3.40 (0.74)	NS	NS	NS
Temporalis middle	3.45 (0.43)	2.83 (1.05)	3.12 (0.83)	NS	NS	NS
Temporalis posterior	2.79 (0.79)	2.67 (0.93)	3.06 (0.87)	NS	NS	NS

CTRL: control group; M-TMD PRE: muscular temporomandibular joint disease group before treatment; M-TMD POST: muscular temporomandibular joint disease group after treatment; NS: not significant.

**Table 2:** Mean scores of OHIP-14, PHQ-9, and GAD-7 in the controlgroup (CTRL) at baseline and in the M-TMD group before and after treatment. All values are expressed as mean (standard deviation).

Questionnaire	CTRL	M-TMD PRE	M-TMD POST	CTRL vs. PRE	CTRL vs. POST	PRE vs. POST
OHIP-14	6.77 (5.85)	20.44 (12.06)	15.18 (11.49)	*p* < 0.05	*p* < 0.05	*p* < 0.05
PHQ-9	6.10 (4.54)	7.14 (5.44)	6.24 (4.56)	*p* < 0.05	NS	*p* < 0.05
GAD-7	4.65 (4.06)	13.79 (5.39)	6.91 (3.42)	*p* < 0.05	NS	*p* < 0.05

GAD-7: General Anxiety Disorder-7 Questionnaire; OHIP-14: Oral Health Impact Profile-14 Questionnaire; PHQ-9: Patent Health Questionnaire-9; NS: not significant.

## Data Availability

The raw data supporting the conclusions of this article will be made available by the authors on request.
